# Photo-Irradiation of Proanthocyanidin as a New Disinfection Technique via Reactive Oxygen Species Formation

**DOI:** 10.1371/journal.pone.0060053

**Published:** 2013-03-20

**Authors:** Keisuke Nakamura, Midori Shirato, Hiroyo Ikai, Taro Kanno, Keiichi Sasaki, Masahiro Kohno, Yoshimi Niwano

**Affiliations:** 1 Laboratory for Redox Regulation, Tohoku University Graduate School of Dentistry, Sendai, Japan; 2 Division of Fixed Prosthodontics, Department of Restorative Dentistry, Tohoku University Graduate School of Dentistry, Sendai, Japan; 3 Department of Bioengineering, Graduate School of Bioscience and Biotechnology, Tokyo Institute of Technology, Yokohama, Japan; University of California, Berkeley, United States of America

## Abstract

In the present study, the bactericidal effect of photo-irradiated proanthocyanidin was evaluated in relation to reactive oxygen species formation. *Staphylococcus aureus* suspended in proanthocyanidin aqueous solution was irradiated with light from a laser at 405 nm. The bactericidal effect of photo-irradiated proanthocyanidin depended on the concentration of proanthocyanidin, the laser irradiation time, and the laser output power. When proanthocyanidin was used at the concentration of 1 mg/mL, the laser irradiation of the bacterial suspension could kill the bacteria with a >5-log reduction of viable cell counts. By contrast, bactericidal effect was not observed when proanthocyanidin was not irradiated. In electron spin resonance analysis, reactive oxygen species, such as hydroxyl radicals, superoxide anion radicals, and hydrogen peroxide, were detected in the photo-irradiated proanthocyanidin aqueous solution. The yields of the reactive oxygen species also depended on the concentration of proanthocyanidin, the laser irradiation time, and the laser output power as is the case with the bactericidal assay. Thus, it is indicated that the bactericidal effect of photo-irradiated proanthocyanidin is exerted via the reactive oxygen species formation. The bactericidal effect as well as the yield of the oxygen radicals increased with the concentration of proanthocyanidin up to 4 mg/mL, and then decreased with the concentration. These findings suggest that the antioxidative activity of proanthocyanidin might prevail against the radical generation potency of photo-irradiated proanthocyanidin resulting in the decreased bactericidal effect when the concentration is over 4 mg/mL. The present study suggests that photo-irradiated proanthocyanidin whenever used in an optimal concentration range can be a new disinfection technique.

## Introduction

Proanthocyanidin also known as procyanidin is a group of polyphenolic compounds naturally occurring in fruits, vegetables, nuts, seeds, and flowers [1], and is a polymer of flavan-3-ol, such as (+)-catechin, (−)-epicatechin, and (−)-epicatechin gallate, with an average degree of polymerization between 2 and 17 [2], [3]. Proanthocyanidin is noteworthy for its antioxidative activity. Since the phenolic hydroxyl group in its structure acts as a hydrogen donor, proanthocyanidin can effectively scavenge free radicals [4], [5]. Besides the antioxidative property, it is suggested that proanthocyanidin has some pharmacological and medicinal properties, such as anti-carcinogenic, anti-inflammatory, and vasodilatory properties [6]. Thus, considerable researches have been conducted to explore therapeutic applications of proanthocyanidin [3], [7].

It is also indicated that proanthocyanidin has antimicrobial and antiviral activities [8]–[11]. An electron microscopic analysis demonstrated that the antimicrobial activity was attributable to a disruption of bacterial cell wall and/or cell membrane [12]. Although the mechanism of antibacterial activity of proanthocyanidin has not been fully elucidated, it would probably be related to the antimicrobial activity of catechins, monomeric units of proanthocyanidin. Concerning the antimicrobial activity of catechins, Arakawa et al. demonstrated that H_2_O_2_ generated in an aqueous solution of epigallocathechin gallate played a critical role in the antimicrobial effect [13]. They proposed the possible mechanism of H_2_O_2_ generation in aqueous solution of catechin by which proton coupled electron transfer to dissolved oxygen resulted in H_2_O_2_ generation. Hence, it is reasonable to consider that antimicrobial mechanism of proanthocyanidin, which consists of polymers of catechins, is similar to that of the monomeric catechins.

The bactericidal activity of H_2_O_2_ can be enhanced by photolysis. In our previous study, it was demonstrated that laser irradiation of H_2_O_2_ at 405 nm could kill bacteria via hydroxyl radical (HO^·^) formation much more effectively than the treatment with H_2_O_2_ alone [14]. It is well-known that HO^·^ possess higher reactivity and oxidative power than H_2_O_2_ [15], [16]. Since it is suggested that H_2_O_2_ is involved in the antibacterial activity of polyphenols, it is expected that the antibacterial activity is enhanced by the laser irradiation. If proanthocyanidin can function as a source of reactive oxygen species (ROS) generation, there would be some merits as a new disinfection technique. For instance, locally injurious properties would be little or nothing because proanthocyanidin is ingested as an ingredient of various fruits, vegetables and functional foods. In fact, proanthocyanidin, which has the highest free radical scavenging effect among various polyphenols [5], might terminate the excessive oxidative damage after the disinfection treatment. Therefore, the purpose of the present study was to evaluate the bactericidal effect of photo-irradiated proanthocyanidin in relation to ROS formation.

## Materials and Methods

### Reagents

Reagents were purchased from the following sources: proanthocyanidin (Leucoselect®) from Indena (Milano, Italy); 4-hydroxy-2,2,6,6-tetramethylpiperidine *N*-oxyl (TEMPOL) from Sigma Aldrich (St. Louis, MO, USA); 5,5-dimethyl-1-pyrroline *N*-oxide (DMPO) from Labotec (Tokyo, Japan); catalase from Wako Pure Chemical Industries (Osaka, Japan); iron (II) sulfate (FeSO_4_) from Kanto Chemical Co. (Tokyo, Japan); and hydrogen peroxide (H_2_O_2_) from Santoku Chemical Industries (Tokyo, Japan). All other reagents used were of analytical grade.

### Preparation of Proanthocyanidin

**Figure 1:**
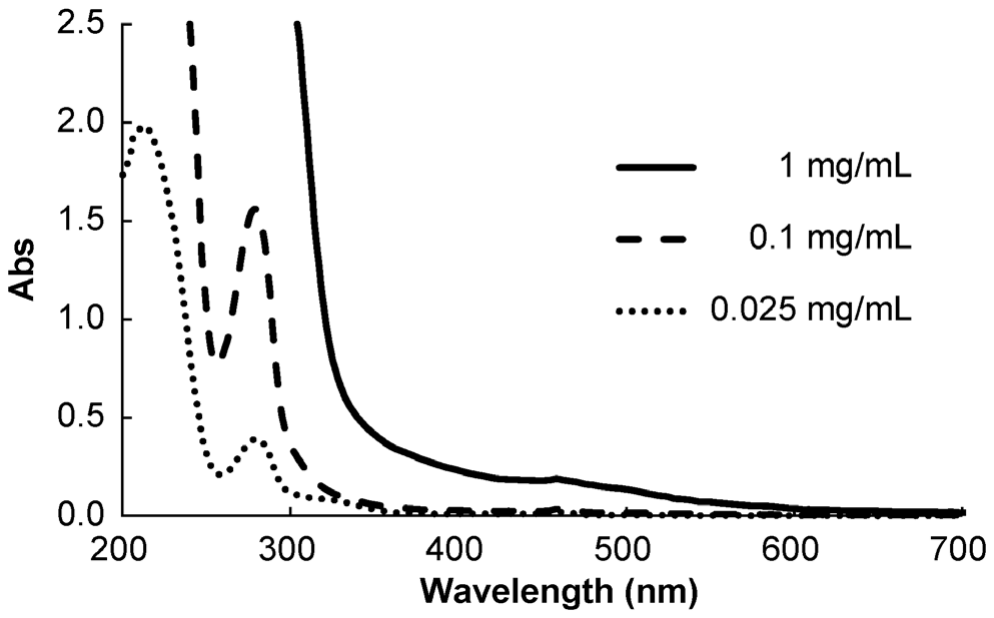
Ultraviolet-visible spectra of proanthocyanidin.

The powder of proanthocyanidin was dissolved in pure water to be 64 mg/mL and then sterilized by filtration. The aqueous solution of proanthocyanidin was prepared at the time of use and was diluted with pure water to given concentrations for each experiment. The ultraviolet (UV)-visible absorption spectra of the 0.025–1 mg/mL proanthocyanidin recorded using a spectrophotometer (Gene Quant 1300; GE Healthcare, Bukinghamshire, UK) are shown in Fig. 1. Since the average molecular weight of proanthocyanidin is estimated to be 1740 [17], 1 mg/mL proanthocyanidin corresponds approximately to 0.6 mM though the precise molecular weight of proanthocyanidin cannot be calculated because it consists of polymers of catechins with different degrees of polymerization.

### Laser Device

A continuous-wave laser device (RV-1000; Ricoh Optical Industries, Hanamaki, Japan) which radiates a light with a wavelength of 405±5 nm was used as a light source according to our previous study [14]. The sample prepared for each experiment was put in a microplate well (96-well) and was irradiated with the laser in a vertical direction. An output power was measured using a laser power meter (FieldMate; Coherent, Santa Clara, CA) to set at 100, 200 and 300 mW. The diameter of the irradiation field was set to equal to that of the well (6.4 mm) so that almost all of the light could pass through the test solution. Thus, the irradiance corresponding to the output power of 100, 200, and 300 mW was calculated to be 310, 620, and 930 mW/cm^2^, respectively.

### Bactericidal Assay

*Staphylococcus aureus* ATCC 25923 purchased from American Type Culture Collection (Manassas, VA) was used. A bacterial suspension was prepared in sterile physiological saline from a culture grown on brain heart infusion (BHI) agar (Becton Dickinson Labware, Franklin Lakes, NJ) aerobically at 37°C for 24 h. In a microplate well, 150 µL of the bacterial suspension was mixed with 150 µL of proanthocyanidin to reach final concentrations of 1×10^7^ cells/mL for the suspension and 0.016–32 mg/mL for proanthocyanidin. Then, the sample was irradiated with the laser-light for 10 min. After the laser irradiation, 50 µL of the sample was mixed with an equal volume of sterile catalase solution (5000 U/mL) to terminate the bactericidal effect of the H_2_O_2_ generated in the proanthocyanidin aqueous solution. A 10-fold serial dilution of the mixture was then prepared using sterile physiological saline, and 10 µL of the diluted solution was seeded onto BHI agar to evaluate the number of viable counts in the suspension. The agar medium was aerobically cultured at 37°C for 24 h and the number of colony forming units (CFU)/mL was determined. The bactericidal effect of photo-irradiated proanthocyanidin [expressed as P(+)L(+)] was compared to the effects of 0.063, 0.5, 4, and 32 mg/mL proanthocyanidin alone [P(+)L(−)], laser irradiation alone [P(−)L(+)], and no treatment [P(−)L(−)]. Thus, P(+) and L(+) stand for sample with proanthocyanidin and laser irradiation of sample, respectively. For the P(−) condition, sterile physiological saline was added to the reaction system instead of proanthocyanidin aqueous solution. For the L(−) condition, the samples were kept in a light-shielding box without laser irradiation. All tests were performed in four independent assays.

In addition, the influence of the laser irradiation time and the laser output power on the bactericidal effect was examined. The bacterial suspension with or without 1 mg/mL proanthocyanidin was treated under the conditions of P(+)L(+), P(+)L(−), and P(−)L(+) for 0, 10, 20, or 30 min. The laser setting was the same as that in the above experiment. The determination of CFU/mL after each treatment was performed as described above. To examine the effect of the laser output power, the bacterial suspension containing 1 mg/mL proanthocyanidin was irradiated with the laser-light at different output power (100, 200, and 300 mW) for 10 min. The determination of CFU/mL after each treatment was performed as described above. All tests were performed in three independent assays.

### Electron Spin Resonance (ESR) Analysis of ROS Generation in Photo-irradiated Proanthocyanidin

Qualitative and quantitative analyses of oxygen radicals, such as HO^·^ and superoxide anion radicals (O_2_^−·^), generated by the laser irradiation of proanthocyanidin were conducted using an ESR spin trapping technique as described in our previous study [18]. In a microplate well, 150 µL of proanthocyanidin dissolved in pure water and 50 µL of DMPO were mixed to reach final concentrations of proanthocyanidin for 0.25–16 mg/mL, and 300 mM for DMPO. Then, the sample was irradiated with the laser-light for 15 s. After irradiation, the sample was transferred to a quartz cell for ESR spectrometry and the ESR spectrum was recorded on an X-band ESR spectrometer (JES-FA-100; JEOL, Tokyo, Japan). The measurement conditions for ESR were as follows: field sweep, 331.4–341.4 mT; field modulation frequency, 100 kHz; field modulation width, 0.05 mT; amplitude, 300; sweep time, 2 min; time constant, 0.03 s; microwave frequency, 9.420 GHz; and microwave power, 4 mW. In each measurement, the ESR spectrum of manganese (Mn^2+^) held in the ESR cavity was recorded and used as an internal standard. To calculate the concentration of HO^·^ spin adduct (DMPO-OH) and O_2_^−·^ spin adduct (DMPO-OOH), the ESR spectrum of 20 µM TEMPOL, a spin-label reagent, was also recorded as a standard. The concentrations of DMPO-OH and DMPO-OOH were determined using Digital Data Processing (JEOL, Tokyo, Japan) by means of comparing the signal intensity of the sample with that of TEMPOL.

The influence of the laser irradiation time and the laser output power on the yield of the oxygen radicals was also examined. The sample containing 1 mg/mL proanthocyanidin and 300 mM DMPO was irradiated with the laser-light at an output power of 300 mW for 0, 5, 10, 15, 30, 60 or 120 s. The ESR analysis for determination of the yield of the oxygen radicals was performed as described above. In the above experiment, it was observed that the yields of DMPO-OH and DMPO-OOH were saturated with the laser irradiation time of >15 s. Two possible causes for the saturation of the spin adducts can be considered; the one is that the generation rates of the oxygen radicals are suppressed, the other is that the formation of the spin adducts is suppressed even though the oxygen radicals are generated constantly. Thus, further experiment was conducted to examine whether the generation rates of the spin adducts or those of the oxygen radicals decrease in relation to the laser irradiation time. For the purpose, 150 µL of proanthocyanidin aqueous solution was irradiated with the laser-light for 120 s prior to the addition of DMPO. After the irradiation, 50 µL of DMPO was added to reach final concentration of 1 mg/mL for proanthocyanidin and 300 mM for DMPO. Immediately after addition of DMPO, the sample was irradiated with the laser-light for 15 s again. Then, the ESR analysis was conducted as described above, and the yields of the oxygen radicals with or without the prior laser irradiation were compared. To examine the effect of the laser output power on the generation of the oxygen radicals, the sample containing 1 mg/mL proanthocyanidin and 300 mM DMPO was irradiated with the laser-light at different output power (100, 200, and 300 mW) for 15 s. The ESR analysis was performed as described above. All tests were performed in three independent assays.

The involvement of dissolved oxygen in the reaction of oxygen radical generation was further examined. One milliliter of 1 mg/mL proanthocyanidin aqueous solution containing 300 mM DMPO put in a glass tube was bubbled with argon gas for 10 min to replace the dissolved oxygen with argon gas. Then, 200 µL of the sample was gently transferred to a microplate well and irradiated with the laser-light at an output power of 300 mW for 30 s. The ESR analysis was performed as described above. All tests were performed in three independent assays.

The yield of H_2_O_2_ was determined utilizing an ESR spin trapping technique coupled with a Fenton reaction, in which H_2_O_2_ reacts with ferrous ions and produces HO^·^. A standard curve was constructed using H_2_O_2_ solutions which contained 1 mg/mL proanthocyanidin. Each standard solution (100 µL) was mixed with 50 µL of DMPO (final concentration: 300 mM) and 50 µL of FeSO_4_ was added to initiate the Fenton reaction. The final concentration of FeSO_4_ was 100 µM. The ESR analysis was performed 30 s after the addition of FeSO_4_ using the same conditions as described above except for the amplitude of 100. Next, the yield of H_2_O_2_ generated by the laser irradiation of proanthocyanidin aqueous solution was evaluated. One hundred microliters of proanthocyanidin aqueous solution (1 mg/mL) in a microplate well was irradiated with the laser-light for 0, 4, 8 or 12 min. Immediately after the irradiation, 50 µL of DMPO and 50 µL of FeSO_4_ were added to the reaction system to reach final concentrations of 300 mM for DMPO and 100 µM for FeSO_4_. DMPO was added to proanthocyanidin aqueous solution after the cessation of laser irradiation so that DMPO trapped only HO^·^ generated by the Fenton reaction. The ESR analysis was performed 30 s after the addition of FeSO_4_ into the reaction system as described above. The yield of DMPO-OH was converted to the yield of H_2_O_2_ using the standard curve. All tests were performed in three independent assays.

### Analysis of Wavelength Dependence of Oxygen Radical Generation

According to the UV-visible absorption spectrum of the 1 mg/mL proanthocyanidin (Fig. 1), the value of absorbance decreased with the increase of wavelength in the range between 300–600 nm. Thus, it was hypothesized that UV light irradiation (<400 nm) generated HO^·^ more efficiently than visible light irradiation if the reaction is caused by the absorbed light energy. To examine wavelength dependence of oxygen radical generation, three light sources with different wavelengths (365, 405 and 532 nm) were used. For the light at 405 nm, the same laser device described above was used while a portable LED UV irradiator (Mos-Cure mini 365; U-Vix, Tokyo, Japan) and an experimental laser device equipped with the second harmonic of Nd-YAG laser (PACT-532-30CW; PAX, Sendai, Japan) were used for the light with wavelengths of 365 and 532 nm, respectively. The output power was measured using the power meter and the irradiance of each light source was set at 30 mW/cm^2^. In a microplate well, 200 µL of 1 mg/mL proanthocyanidin containing 300 mM DMPO was irradiated for 120 s using each light source. After irradiation, the ESR analysis was performed as described above. Furthermore, the absorbance of 1 mg/mL proanthocyanidin at wavelengths of 365, 405, and 532 nm was analyzed using the spectrophotometer. All tests were performed in three independent assays.

### Statistical Analyses

Statistical significance (p<0.05) in the CFU/mL obtained in the bactericidal test with different concentrations of proanthocyanidin was assessed by Dunnett’s multiple comparison test using the initial bacterial count as a control group. The time-course changes in the CFU/mL under the conditions of P(+)L(+), P(+)L(−), and P(−)L(−) and the effect of laser output power on the bactericidal effect of P(+)L(+) were assessed by two-way ANOVA (analysis of variance) and Tukey-Kramer multiple comparison test, respectively. All the analyses for the bactericidal test were performed following logarithmic conversion. Statistical significances (p<0.05) in the yields of DMPO-OH and DMPO-OOH with the laser irradiation at different laser output powers, the yields with or without the prior laser irradiation, the yields with or without argon gas replacement, the yields with the irradiation at different wavelength of light, and the absorbance of proanthocyanidin at 365, 405, and 532 nm were assessed by Student’s *t*-test for pairwise comparisons and Tukey-Kramer test for multiple comparisons.

## Results

### Bactericidal Assay

**Figure 2:**
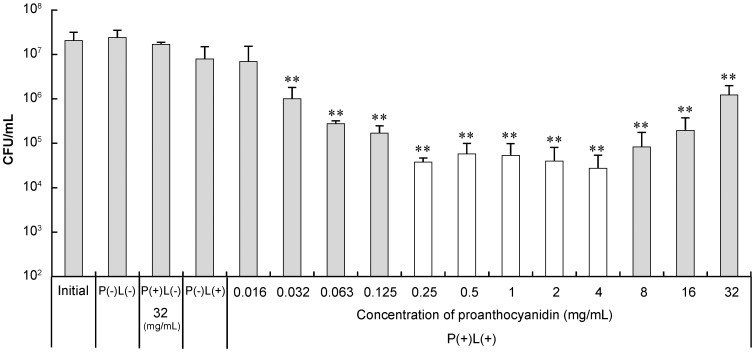
Bactericidal effect of photo-irradiated proanthocyanidin in relation to the concentration. For the L(+) condition, the laser irradiation was performed at an output power of 300 mW for 10 min. Each value is the mean of four independent measurements with the standard deviation. Significant differences from the initial bacterial count are shown as p<0.01 (**).

When proanthocyanidin used at a concentration of 0.032 mg/mL or more was irradiated with the laser-light, the CFU/mL significantly decreased from the initial bacterial count (Fig. 2). The bactericidal effect of photo-irradiated proanthocyanidin against *S. aureus* increased with the concentration up to 0.25 mg/mL, reached a plateau up to 4 mg/mL and then decreased with the concentration at 8 mg/mL or higher. Thus, the maximum bactericidal effect was obtained in the range of concentration between 0.25 and 4 mg/mL. When proanthocyanidin was used at the concentration of 0.25–4 mg/mL, the bacteria were killed under the condition of P(+)L(+) with a >2.5-log reduction of the CFU/mL in 10 min-treatment. By contrast, the conditions of P(+)L(−), P(−)L(+), and P(−)L(−) showed no significant difference in the CFU/mL from the initial bacterial count within the 10 min-treatment (Fig. 2). Under the condition of P(+)L(−), the bacteria were not killed by the proanthocyanidin at any concentrations tested (data not shown).

**Figure 3:**
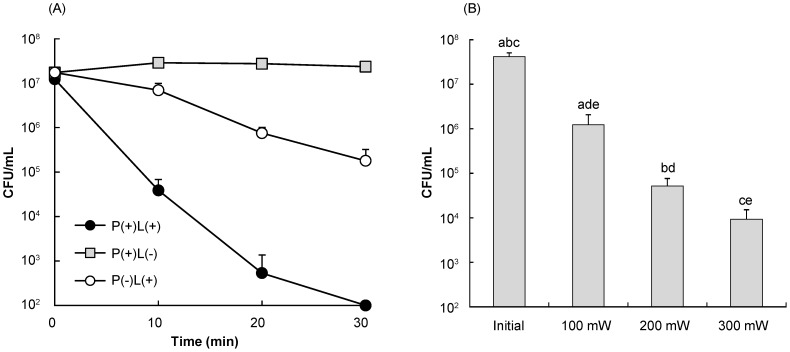
The influence of laser irradiation time and laser output power on the bactericidal effect of photo-irradiated proanthocyanidin. (A) Time-course changes in CFU/mL under the conditions of P(+)L(+), P(−)L(+)and P(+)L(−). For the P(+) condition, 1 mg/mL proanthocyanidin was used. For the L(+) condition, laser output power was set at 300 mW. (B) Bactericidal activity of 1 mg/mL proanthocyanidin irradiated with the laser-light at different output powers. Each value is the mean of three independent measurements with the standard deviation. Significant differences between the conditions are demonstrated by the same alphabetic letters. p<0.05 (a), p<0.01 (b, c, d, e).

The bacteria could be killed in a laser irradiation time dependent manner under the conditions of P(+)L(+) and P(−)L(+) (Fig. 3A). However, the bactericidal effect of the P(+)L(+) was higher than that of P(−)L(+) at each time point. The laser irradiation of 1 mg/mL proanthocyanidin killed the bacteria with a >5-log reduction of CFU/mL in 30 min. On the other hand, the laser irradiation alone for 30 min killed the bacteria with a <2-log reduction. The 1 mg/mL proanthocyanidin without the laser irradiation [P(+)L(−)] did not show bactericidal effect within 30 min. The two-way ANOVA showed that the treatment conditions, time, and combination of treatment conditions and time significantly affected the reduction of CFU/mL (Table 1). The laser output power also affected the bactericidal effect of photo-irradiated proanthocyanidin. When the bacterial suspension was irradiated for 10 min with the laser at an output power of 100, 200, and 300 mW, approximately 1-, 2-, and 3-log reduction of viable cell counts were obtained (Fig. 3B). There were significant differences in the CFU/mL among the conditions except for the conditions irradiated at 200 and 300 mW of which p-value was 0.069.

**Table 1:** ANOVA summary table for the time-course changes in the CFU/mL in each treatment group.

	Sum of squares	df	Mean square	F value	P value
Treatment	48.24	2	24.12	33.3207	<0.0001
Time	13.53	2	6.76	9.3439	0.0016
Treatment*Time	15.22	4	3.81	5.2574	0.0055
Error	13.03	18	0.72		

df: degree of freedom.

### ESR Analysis of ROS Generation in Photo-irradiated Proanthocyanidin

**Figure 4:**
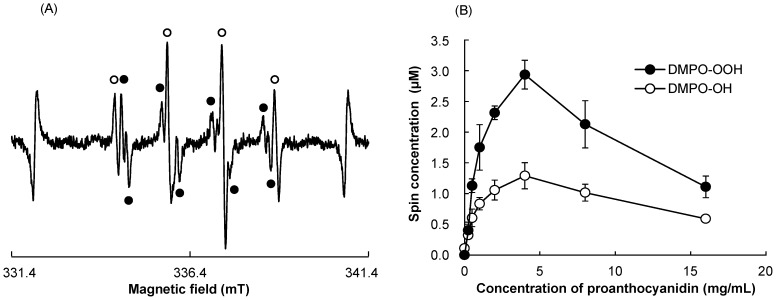
ESR-spin trapping analysis for hydroxyl radicals and superoxide anion radicals generated in photo-irradiated proanthocyanidin aqueous solution. (A) Representative ESR signals of DMPO-OH (open circle) and DMPO-OOH (solid circle) obtained when proanthocyanidin aqueous solution was irradiated with the laser-light for 15 s. (B) The yields of DMPO-OH and DMPO-OOH after given concentrations of proanthocyanidin were irradiated with the laser-light for 15 s. Each value is the mean of three independent measurements with the standard deviation.

When proanthocyanidin aqueous solution containing DMPO was irradiated with the laser-light, the ESR signals of DMPO-OH and DMPO-OOH were detected (Fig. 4A). The presence of the two spin adducts were confirmed by hyper fine coupling constants of a_N_ = a_H_ = 1.49 mT for DMPO-OH and a_N_ = 1.41 mT, a_Hα_ = 1.13 mT, and a_Hβ_ = 0.13 mT for DMPO-OOH [19]. The yields of DMPO-OH and DMPO-OOH changed dependently on the concentration of proanthocyanidin (Fig. 4B). When the concentration of proanthocyanidin was up to 4 mg/mL, the yield of DMPO-OH and DMPO-OOH increased with the concentration. Then, the yields decreased with the concentration when the concentration of proanthocyanidin was higher than 4 mg/mL (Fig. 4B).

**Figure 5:**
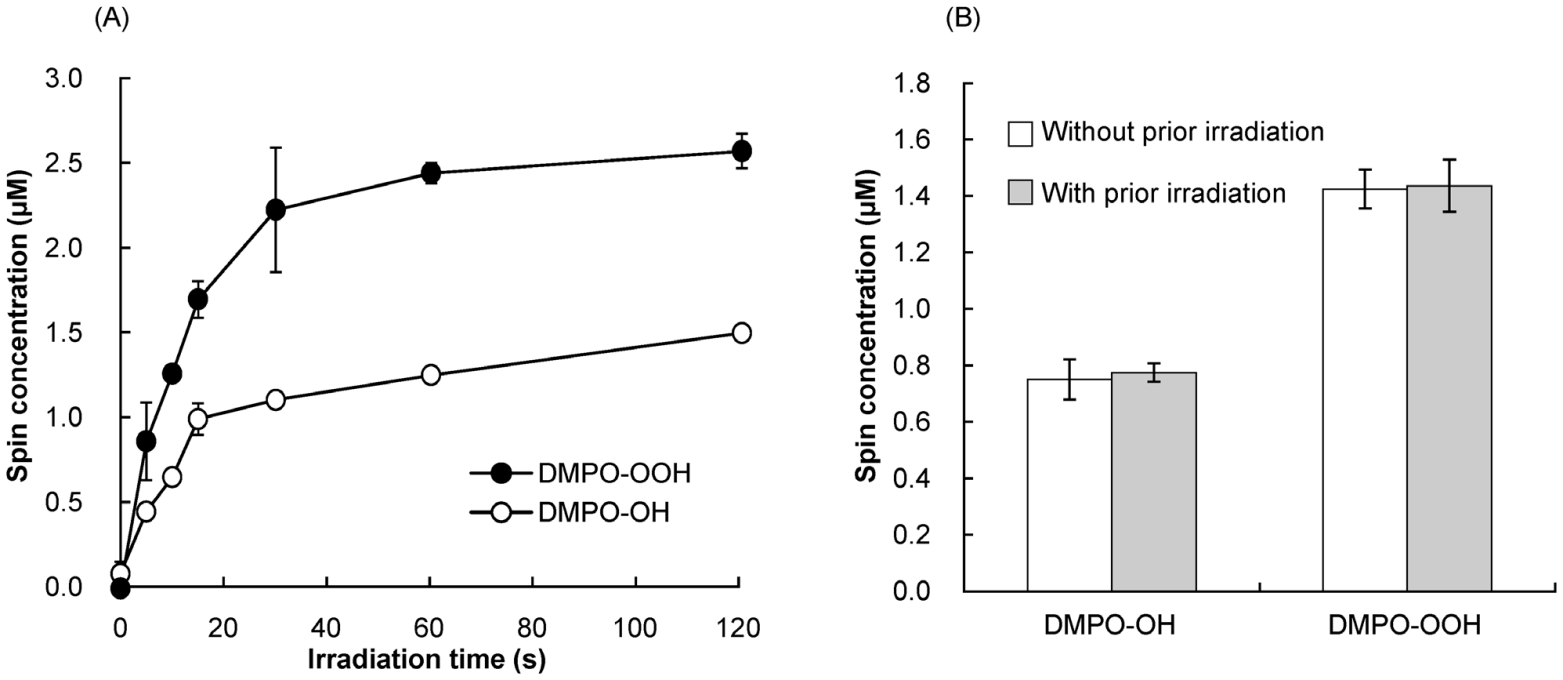
The influence of laser irradiation time on the yields of DMPO-OH and DMPO-OOH. (A) Time-course changes in the yields of DMPO-OH and DMPO-OOH when 1 mg/mL proanthocyanidin was irradiated at an output power of 300 mW. (B) The comparison of the yields of DMPO-OH and DMPO-OOH generated in the photo-irradiated proanthocyanidin with or without the prior laser irradiation. There were no statistically significant differences between the two groups. Each value is the mean of three independent measurements with the standard deviation.

**Figure 6:**
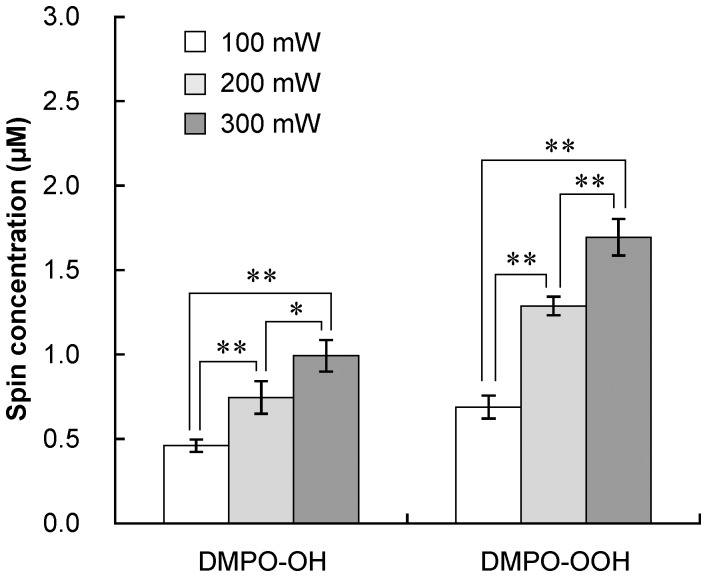
The influence of laser output power on the yields of DMPO-OH and DMPO-OOH. Proanthocyanidin (1 mg/mL) was irradiated with the laser-light for 15 s. Each value is the mean of three independent measurements with the standard deviation. Significant differences between the conditions are shown as p<0.05 (*) and p<0.01 (**).

**Figure 7:**
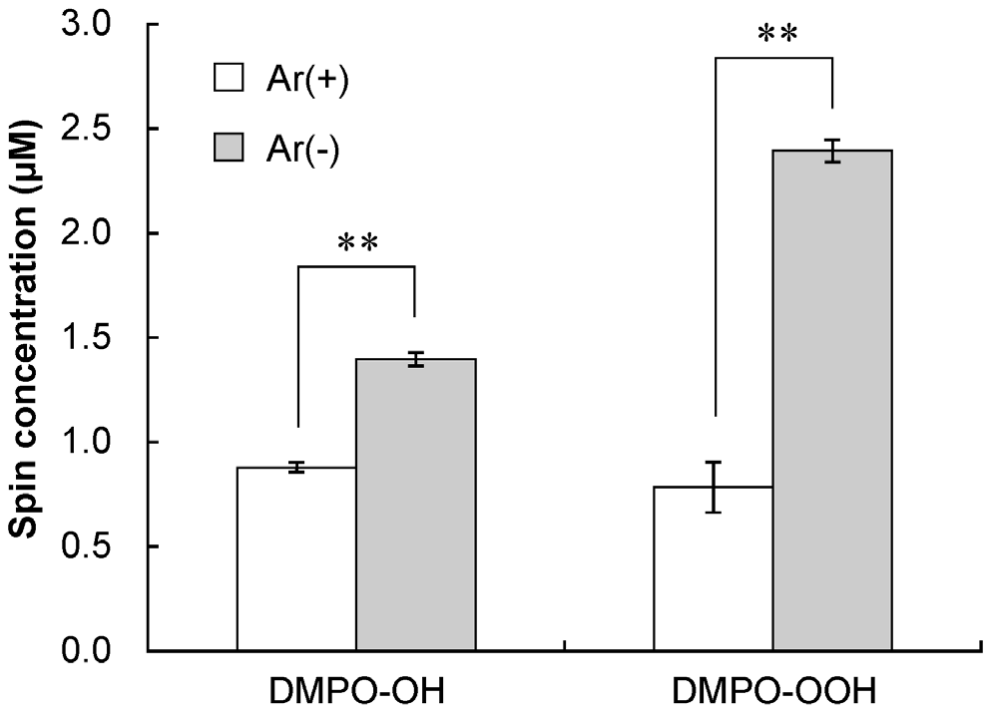
The yield of DMPO-OH and DMPO-OOH generated by photo-irradiation of 1 mg/mL proanthocyanidin with or without argon gas replacement. The laser irradiation was performed at an output power of 300 mW for 30 s. Each value is the mean of three independent measurements with the standard deviation. Significant differences between the conditions are shown as p<0.01 (**).

The yields of DMPO-OH and DMPO-OOH in the photo-irradiated proanthocyanidin (1 mg/mL) increased with the laser irradiation time (Fig. 5A). When the proanthocyanidin aqueous solution was irradiated with the laser-light for 15 s, the yields of DMPO-OH and DMPO-OOH were 0.99 and 1.69 µM, respectively. However, the generation rates of the DMPO-OH and DMPO-OOH decreased with the irradiation time of >15 s. Thus, further experiment was conducted to examine if the prior laser irradiation of proanthocyanidin affected the generation rate of the oxygen radicals. There was no significant difference in the yields of the spin adducts generated in the photo-irradiated proanthocyanidin with or without the prior laser irradiation (Fig. 5B), suggesting that HO^·^ and O_2_^−·^ are continuously generated even though the yields of DMPO-OH and DMPO-OOH were saturated when proanthocyanidin was irradiated for >15 s. Besides the laser irradiation time, the laser output power also significantly affected the yields of DMPO-OH and DMPO-OOH, and the yields increased with the output power (Fig. 6). Furthermore, it was demonstrated that replacement of dissolved oxygen in proanthocyanidin solution with argon gas significantly decreased the yield of both oxygen radicals (Fig. 7).

**Figure 8:**
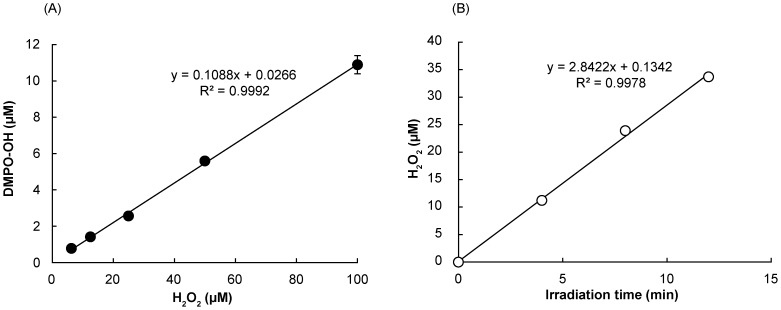
Quantification of H_2_O_2_ generated in photo-irradiated proanthocyanidin aqueous solution. (A) Standard curve for the analysis. (B) The yield of H_2_O_2_ in the 1 mg/mL proanthocyanidin aqueous solution when irradiated with the laser at an output power of 300 mW. Each value is the mean of three independent measurements with the standard deviation.

As shown in Fig. 8A, the concentration of H_2_O_2_ highly correlated with the yield of DMPO-OH generated in the Fenton reaction. Based on the standard curve, H_2_O_2_ generation by the laser irradiation of 1 mg/mL proanthocyanidin was analyzed. The yield of H_2_O_2_ linearly increased with the laser irradiation time and the average yield of H_2_O_2_ after 12 min laser irradiation was 33 µM (Fig. 8B).

### Analysis of Wavelength Dependence of Oxygen Radical Generation

**Figure 9:**
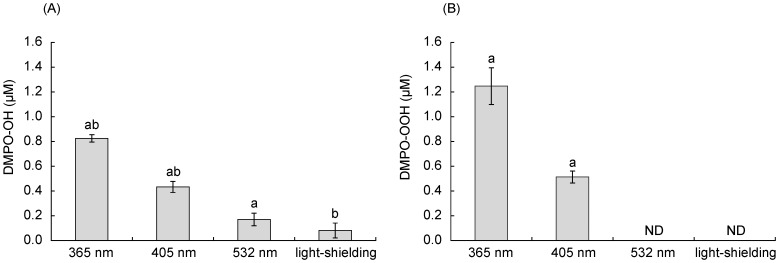
The yield of DMPO-OH and DMPO-OOH generated by photo-irradiation of 1 mg/mL proanthocyanidin at different wavelengths. Photo-irradiation at each wavelength was performed at irradiance of 30 mW/cm^2^ for 120 s. Each value is the mean of three independent measurements with the standard deviation. Significant differences between the conditions are demonstrated by the same alphabetic letters. p<0.01 (a, b). ND: not detected.

**Figure 10:**
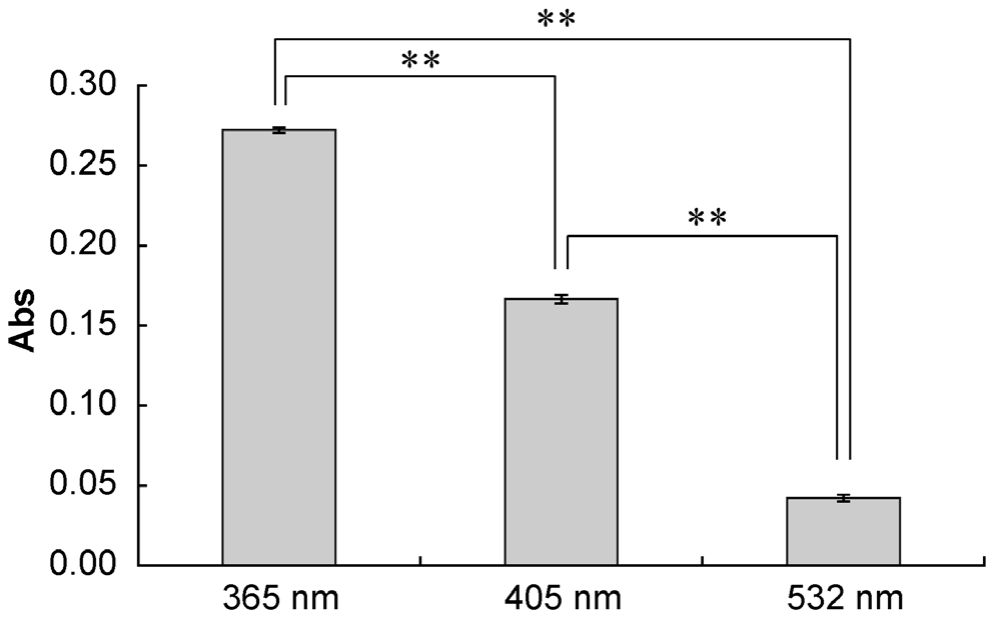
Absorbance of 1 mg/mL proanthocyanidin at each wavelength. Each value is the mean of three independent measurements with the standard deviation. Significant differences in the absorbance at given wavelengths are shown as p<0.01 (**).

The yields of DMPO-OH and DMPO-OOH in photo-irradiated proanthocyanidin were significantly affected by the wavelength of light (Fig. 9A, B). When the proanthocyanidin was irradiated with the light at 365 nm, the yields of both oxygen radicals were approximately two-fold of those generated by irradiation at 405 nm. By contrast, the irradiation at 532 nm generated no or little amount of both oxygen radicals. In addition, there were significant differences in the absorbance of 1 mg/mL proanthocyanidin at 365, 405, and 532 nm (Fig. 10).

## Discussion

The present study demonstrated for the first time that proanthocyanidin could exert bactericidal activity against *S. aureus* when irradiated with the laser-light at 405 nm. The bactericidal effect of photo-irradiated proanthocyanidin increased with the laser irradiation time resulting in a >5-log reduction of the CFU/mL within 30 min. By contrast, proanthocyanidin treatment without laser irradiation did not exert bactericidal effect. Although the antimicrobial activity of proanthocyanidin alone was reported in previous studies [9], [10], [20], it is known that the activity is moderate and it takes long time, at least several hours, to kill *S. aureus* with a >5-log reduction [8]. Although the laser irradiation performed in the present study could kill the bacteria, the bactericidal effect (<2-log reduction of the CFU/mL in 30 min) was weaker than that of photo-irradiated proanthocyanidin. This finding agrees with a previous study, which showed that irradiation with UV or visible blue light could exert bactericidal action depending on the level of irradiation [21]. Even though treatment with proanthocyanidin or laser irradiation alone was not effective, the laser irradiation of the suspension containing proanthocyanidin could kill the bacteria very effectively.

The ESR analysis demonstrated that HO^·^ and O_2_^−·^ were generated when the proanthocyanidin aqueous solution was irradiated with the laser-light. The yields of both oxygen radicals depended on the concentration of proanthocyanidin. Bell-shaped yields were obtained for both oxygen radicals, and the highest yields were observed when the proanthocyanidin was used at the concentration of 4 mg/mL. This would be attributable to the strong antioxidative activity of proanthocyanidin [22], [23]. When the concentration is over 4 mg/mL, the antioxidative activity might prevail against the radical generation potency of photo-irradiated proanthocyanidin resulting in the decreased yields. Interestingly, there was also an optimal concentration range of proanthocyanidin, in which the bacteria were killed effectively. The range was from 0.25 to 4 mg/mL, and the yields of oxygen radicals also increased up to the concentration of 4 mg/mL in the ESR analysis. Thus, it is considered that the optimal concentration range would probably be determined by the relationship between antioxidative and oxidative activity of photo-irradiated proanthocyanidin.

The other results of the ESR analysis indicate that HO^·^ and O_2_^−·^ are continuously generated with the laser irradiation time even though the yields of DMPO-OH and DMPO-OOH are saturated, and the yields of HO^·^ and O_2_^−·^ increased with the laser output power. In addition, H_2_O_2_ was generated in a time dependent manner when proanthocyanidin aqueous solution was irradiated with laser. Since the bactericidal effect of photo-irradiated proanthocyanidin also increased with the laser irradiation time and the laser output power, the bactericidal effect is most likely exerted in relation to the ROS generation. When the oxidative power of each ROS is taken into account [15], HO^·^ is probably a main contributor to the bactericidal action. O_2_^−·^ would be generated as a result of oxidation of proanthocyanidin initiated by the photo-irradiation in the presence of dissolved oxygen, and O_2_^−·^ would further be reduced to H_2_O_2_ as is the case of autoxidation of catechins [24]. Then, H_2_O_2_ would be photolyzed by the laser irradiation resulting in the generation of HO^·^ [14], [25].

Although proanthocyanidin does not have the absorption peak around 365–532 nm, it absorbs the light somehow in the range of those wavelengths. There were significant differences in the absorbance at 365, 405, and 532 nm. Based on this finding, further analysis was conducted to examine if the yields of oxygen radicals depended on wavelength of light source. The result showed that UV light irradiation generated both HO^·^ and O_2_^−·^ more efficiently than visible light irradiation. These findings strongly suggest that the reaction of oxygen radical generation via photo-irradiation of proanthocyanidin is originally derived from photo-oxidation in which the light energy absorbed by proanthocyanidin triggers the oxidation utilizing dissolved oxygen. This reaction is a photo-chemical reaction and can be designated as a photo-oxidation.

If the generation rate of HO^·^ obtained in the ESR analysis (0.99 µM/15 s) is constant independently of the irradiation time as suggested in the present study, the yield of HO^·^ in 30 min would be 120 µM in total. This was lower than the amount of HO^·^ needed to kill *S. aureus* with a >5-log reduction in our previous study [14]. Our earlier results suggested that 200–300 µM HO^·^ would be needed to kill *S. aureus* with a >5-log reduction. Nonetheless, the photo-irradiated proanthocyanidin could kill the bacteria with a >5-log reduction. One of the reasons might be because of the affinity of proanthocyanidin to bacterial cell membrane. Epicatechin gallate and epigallocatechin gallate, monomeric units of proanthocyanidin, possess the affinity to cell membranes [26], [27]. In addition, Ikigai et al. demonstrated that the antimicrobial activity of epigallocatechin gallate was higher than that of epicatechin, and showed that epigallocatechin gallate but not epicatechin damaged the lipid bilayers of liposomes [28]. Thus, proanthocyanidin would also have the affinity to cell membrane because of pyrogallol moiety in its polymeric structure. Consequently, the HO^·^ generated around the bacterial cell membrane will affect the membrane structure and function. Besides the ROS, other radical species such as semiquinone radicals might be involved in the photo-oxidation of proanthocyanidin [29], and as such they might contribute to the bactericidal activity. The involvements of the semiquinone radicals and other compounds in the bactericidal activity should be addressed in the future study.

The results of the present study suggest that the laser irradiation of proanthocyanidin at 405 nm can be used as a new disinfection technique. The goal of the present study was to develop a new disinfection technique which can be applied to the medical field and food sanitation. Since the UV irradiation damages living body and food, we selected visible blue light (405 nm) to avoid excessive damage caused by irradiation alone even though UV irradiation generated oxygen radicals more effectively. Since proanthocyanidin functions as both antioxidant and pro-oxidant, it is crucial to regulate both characteristics in terms of utilizing the technique for the disinfection treatment. During the disinfection treatment, photo-irradiated proanthocyanidin generates ROS to kill bacteria, and then proanthocyanidin can serve as an antioxidant after cessation of laser irradiation. More in detail, since the generation of the oxygen radicals from proanthocyanidin is terminated by cessation of laser irradiation, the oxidative effect does not continue after the disinfection treatment. Even if excessive amount of the oxygen radicals would be generated, they cannot exist for long time because their life time is extremely short [16], [30]. Concerning H_2_O_2_, it might remain in the disinfection solution even after the laser irradiation of proanthocyanidin but the concentration is much lower than that used as a disinfectant, 3 wt/vol % corresponding to 880 mM, and a small amount of HO^·^ derived from H_2_O_2_ could be sufficiently scavenged by proanthocyanidin. Thus, the residual toxicity is practically negligible. Therefore, it is expected that this disinfection technique is applicable to the fields of medical and food sanitation. For further development of the disinfection technique, the bactericidal effect against biofilm, which generally shows high resistance to antibiotics and disinfectant, should be evaluated.
